# A comparison of the functional modules identified from time course and static PPI network data

**DOI:** 10.1186/1471-2105-12-339

**Published:** 2011-08-15

**Authors:** Xiwei Tang, Jianxin Wang, Binbin Liu, Min Li, Gang Chen, Yi Pan

**Affiliations:** 1 School of Information Science and Engineering, Central South University, Changsha, 410083, China; 2 School of Information Science and Engineering, Hunan First Normal University, Changsha, 410205, China; 3 Department of Computer Science, Georgia State University, Atlanta, GA 30302-4110, USA

## Abstract

**Background:**

Cellular systems are highly dynamic and responsive to cues from the environment. Cellular function and response patterns to external stimuli are regulated by biological networks. A protein-protein interaction (PPI) network with static connectivity is dynamic in the sense that the nodes implement so-called functional activities that evolve in time. The shift from static to dynamic network analysis is essential for further understanding of molecular systems.

**Results:**

In this paper, Time Course Protein Interaction Networks (TC-PINs) are reconstructed by incorporating time series gene expression into PPI networks. Then, a clustering algorithm is used to create functional modules from three kinds of networks: the TC-PINs, a static PPI network and a pseudorandom network. For the functional modules from the TC-PINs, repetitive modules and modules contained within bigger modules are removed. Finally, matching and GO enrichment analyses are performed to compare the functional modules detected from those networks.

**Conclusions:**

The comparative analyses show that the functional modules from the TC-PINs have much more significant biological meaning than those from static PPI networks. Moreover, it implies that many studies on static PPI networks can be done on the TC-PINs and accordingly, the experimental results are much more satisfactory. The 36 PPI networks corresponding to 36 time points, identified as part of this study, and other materials are available at http://bioinfo.csu.edu.cn/txw/TC-PINs.

## Background

Over the past decade, most research on biological networks has been focused on static topological properties, describing networks as collections of nodes and edges. Computational analysis of these networks has great potential in aiding our understanding of gene function, biological pathways and cellular organization. But, in reality, cellular systems are highly dynamic and responsive to cues from the environment [1]. Cellular function and response patterns to external stimuli are regulated by biological networks, such as PPI, metabolic, signaling, transcription regulatory networks and neural synapses. Such networks are representations of large-scale dynamic systems. While significant progress has been made in computational analysis of proteome-scale cellular networks, the dynamics inherent within these networks are often overlooked in computational network analysis. Since there typically is little direct information available on the temporal dynamics of these network interactions, the majority of molecular interaction network modeling and analysis have been solely focused on static properties. However, proper cellular functioning requires the precise coordination of a large number of events and identifying the temporal and contextual signals underlying proposed interactions is a crucial part of understanding cellular function. Network maps are graphical representations of dynamic systems in life. A network with a static connectivity is dynamic in the sense that the nodes implement so-called functional activities evolving in time. In a biological context, these activities may represent the concentration of a molecule, the phosphorylation state of an enzyme, the expression level of a gene, or the depolarization of a neuron or circadian rhythm.

The moment has now come when the shift from static to dynamic network analysis is essential for further understanding of molecular systems. One of the very first things is to determine what we mean by interaction or network 'dynamics'. In simple terms, whether an interaction occurs or not depends upon spatial, temporal and/or contextual variation. Interactions may be constitutive or obligate, or may instead occur only in specific situations. Among these dynamically varying interactions (sometimes referred to as transient interactions), the variation may be either reactive (i.e., caused by exogenous factors, such as a response to some environmental stimulus) or programmed (i.e., due to endogenous signals, such as cell-cycle dynamics or developmental processes). Contextual variation overlaps heavily with temporal variation, but focuses more specifically on characterizing reactive variation and the conditions that cause it. Studying context may also encompass examining sequence or genetic variation within a population of contemporaries and exploring how that variation affects network interactions, topology and function [2]. When development, disease progression and cyclical biological processes, e.g., the cell cycle, metabolic cycle [3] and even entire life cycles, are studied, time course analysis becomes an important tool. Recent research efforts have considered using static measurements to 'fill in the gaps' (the *gaps *refer to accurate temporal parameters that are not yet available for many protein-protein interactions) in the time series data [4], quantifying timing differences in gene expression and reconstructing regulatory relationships. By integrating yeast PPI networks with gene expression data, Han *et al*. suggested that some modules are active at specific times and locations [5]. In a study that described dynamic protein complex formation during cell cycles, it was found that constitutively expressed and cell cycle-regulated proteins form protein complexes together at particular time points during the cell cycle [6]. Qi *et al*. further noted that the integration of a variety of datasets, including binary interactions, protein complexes and expression profiles enables the identification of subnetworks that are active under certain conditions [7]. Here we focus on the temporal aspects of networks, which allow us to study the dynamics of protein module assembly during the *S. cerevisiae *cell cycle. Although accurate temporal parameters are not yet available for PPI systems, by integrating additional biological resources that contain such information (e.g., gene expression data), people can solve or partially solve this problem. In this paper, Time Course Protein Interaction Networks (TC-PINs) are reconstructed by incorporating time series gene expression data [3] into a PPI network (http://dip.doe-mbi.ucla.edu/dip/Download.cgi?SM = 7).

Because we have unfolded a static PPI network in time (dynamics), it will be necessary to make a principal distinction between two biological concepts, namely, protein complexes and functional modules. A protein complex is a physical aggregation of several proteins (and possibly other molecules) via molecular interaction (binding) with each other at the same location and time. A functional module also consists of a number of proteins (and other molecules) that interact with each other to control or perform a particular cellular function. However, unlike protein complexes, these proteins do not necessarily interact at the same time and location [8]. Song *et al*. utilized an external measure - the Gene Ontology(GO) [9] - to define functional modules [10]. That is, for a GO biological process or cellular component functional term, the corresponding module contains all the proteins that are annotated with that term.

After the TC-PINs are constructed, a representative clustering algorithm [11] is selected and used to create functional modules from the TC-PINs. Then repetitive modules and those modules that are contained in bigger modules are removed. The same method used by Bader *et al*. [12] is also used to determine how effectively the remaining modules match the known modules. To have a further understanding of biological significance of the modules, Gene Ontology enrichment analysis is performed. Finally, as the point of reference, the clustering algorithm also uncovers modules from the static PPI network and the pseudorandom network and analyses of these similar results are underway.

## Method

### Datasets

The DIP (Database of Interacting Proteins) database lists protein pairs that are known to interact with each other. The interaction indicates that two amino acid chains are experimentally identified to bind to each other. The database lists such pairs to aid those studying a particular PPI, but it also aids those investigating entire regulatory and signaling pathways, as well as those studying the organization and complexity of the PIN at the cellular level. The PPI data of *S. cerevisiae *used in this work is from DIP (http://dip.doe-mbi.ucla.edu/dip/Download.cgi?SM = 7/), updated on Oct. 10, 2010. The static yeast PPI network includes 4,950 distinct proteins and 21,788 interactions totally. As is customary, self interactions representing autoregulation or protein homodimerization are not included in the analysis. Furthermore, duplicated interactions are ignored.

Time course gene expression data and periodic transcripts data of *S. cerevisiae *are from [3], updated on Apr 14, 2011. Raw microarray data are also available from Gene Expression Omnibus, accession number GSE3431. The dataset, in the form of a 9,335 × 36 matrix, includes expression profiles of 9,335 probes under 36 different time points. We map probe sets to gene symbols according to the annotation file provided by Affymetrix and thus obtain 6,777 budding yeast *S. cerevisiae *gene products. The periodic transcripts file contains data for 3552 unique expressed genes that are periodic with at least 95% confidence, which corresponds to 3656 probes [3].

Gene ontologies and annotations used in GO enrichment analysis are downloaded from http://geneontology.org (http://www.geneontology.org/gene-associations/submission/), updated on July 24, 2010.

### Reconstruction of the TC-PINs

Before that, the first issue, perhaps, is to determine the consistency of both datasets selected. Upon comparing the 4,950 proteins extracted from a static PPI network (http://dip.doe-mbi.ucla.edu/dip/Download.cgi?SM=7) with 6,777 gene products from gene-expressing profiles [3], we find that they share 4,858 proteins. Thus, the gene-expressing profiles can cover more than 98% of the proteins in the static PPI network. In other words, the result shows that it is reasonable to combine the two datasets.

Then, a bigger challenge is how to choose an appropriate cutoff threshold in order to filter the gene expression profiles and retain merely the most biologically significant gene products. This threshold application step is a major juncture in which errors can be introduced in the form of both false negatives and false positives. By setting this threshold too high, important gene products can be lost. Similarly, we must be sure to remove gene products that have no apparent biological significance. Some of the methods that have been applied to the threshold selection problem in various types of networks include using an arbitrary threshold [13], retaining only the top *x *percent of the strongest relationships [14], permutation testing [15] and filtering based upon control spot correlations [16] or the statistical significance of the relationships [17,18].

Tu *at al*. [3] used a continuous culture system to reveal a robust, metabolic cycle in budding yeast. Each cycle was characterized by a reductive, nonrespiratory phase followed by an oxidative, respiratory phase wherein the synchronized culture rapidly consumed molecular oxygen. After performing microarray analysis of gene expression, they found that over half of yeast genes (~ 3552) exhibited periodic expression patterns at a confidence level of 95% over three consecutive yeast metabolic cycle (12 time intervals per cycle). 1023 periodic genes encoding ribosomal proteins, translation initiation factors, amino acid biosynthetic enzymes, small nuclear RNAs, RNA processing enzymes and proteins required for the uptake and metabolism of sulfur exhibit a similar expression pattern of peaking in the Ox(oxidative) metabolism. 977 periodic genes during the R/B (reductive/building) metabolism peak when cells begin to cease oxygen consumption. This set consists primarily of nuclear encoded mitochondrial genes as well as genes encoding histones, spindle pole components and proteins required for DNA replication and cell division. 1510 genes expressed maximally in the R/C (reductive/charging) metabolism encode proteins involved in nonrespiratory modes of metabolism and protein degradation. These periodic genes play an important role in yeast metabolic cycle, so they have biological significance. Moreover, Tu *at al *[3] also indicate that the average expression levels of periodic transcripts is 1.7-fold higher than that of non-periodic transcripts. After looking into the expressing peak value of every periodic gene during one cycle (12 time intervals), we discover that about 82% periodic genes have expression peak value more than 1.6.

Therefore, to select a large number of periodic genes, we take a similar tactic as used by Ala *et al*. [14] to determine a potential threshold value. That is, for every time point, we set a fix threshold to filter the transcripts. Only the transcripts whose expression levels are higher than the threshold value will be remained.

Our algorithm for reconstructing the TC-PINs is structured as sequence of following steps:

(i) Filtering gene expressing profiles

The approach we use to filter the raw gene expressing data is by comparing the expression levels of genes at every time points to a fixed threshold, for example 0.7. How the cut-off is chosen is discussed in *Effect of the threshold selection*. After filtering the gene expression profiles, about 56.78% raw transcripts remain. 36 different gene product sets are obtained at 36 time points. Tu *et al*. [3] classified raw probes that had at least 3 (on average one per cycle) present (P) calls (as generated by Affymetrix GeneChip software) as expressed. According to this criterion, out of 9,335 probes queried by the YG_S98 array, 7,985 (about 86%) are expressed. Out of 6,555 probes querying unique, annotated open reading frames (ORFs), 6,209 are expressed. By filtering the gene expressing profiles, around 43% raw transcripts with low expressing levels are removed and the gene products that have no apparent biological significance are basically discarded.

(ii) Reconstruction of the TC-PINs

If two interacting proteins in the static PPI network also present in the gene product set at a certain time point, the two proteins and their interaction form a part of a TC-PIN at the time point. The process is repeated until the TC-PIN is created. Similarly, 36 TC-PINs can be reconstructed.

**Figure 1:**
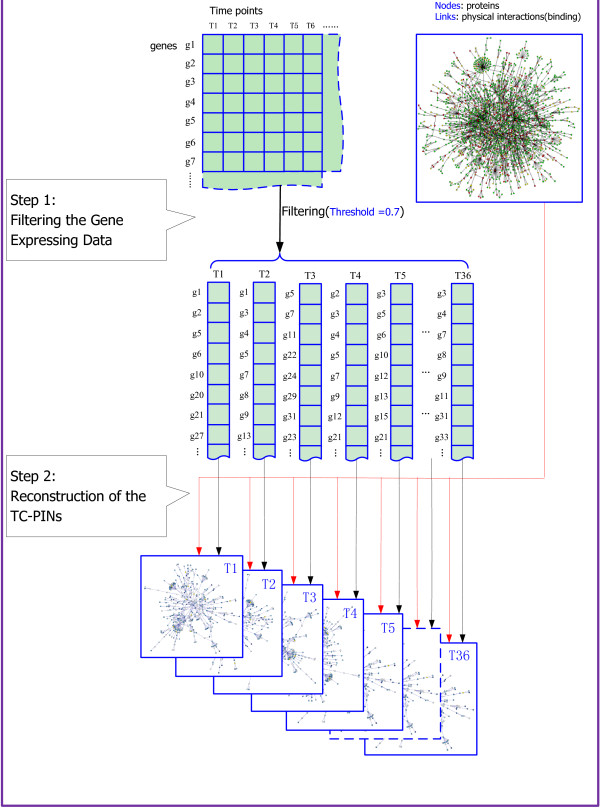
**Schematic overview of reconstruction of the TC-PINs. Our method mainly consists of two stages, screening gene expressing profiles and reconstructing the TC-PINs**. *Reconstructing the TC-PINs*. Input: static PPI network *G *= (*V*,*E *) and sets of gene products *S1*, *S2*, · · ·, *S36 *; Output: the TC-PINs; 1. For Each pair of interacting proteins(*pi*, *pj *) in G, do *pi ∈ S1 *and *pj ∈ S1 *then the protein interaction pair is selected as part of TC-PIN *S1*. 2. Repeating step 1, until all interactions in static PPI network have been processed. As a result, TC-PIN *S1 *has been reconstructed. 3. Similarly, remaining TC-PINs can also be generated.

Figure 1 shows how to reconstruct the TC-PINs. Our method mainly consists of two stages, screening gene expressing profiles and reconstructing the TC-PINs.

### Identifying functional modules from the TC-PINs

The next urgent task is to identify meaningfully functional modules from the TC-PINs. So far, the Markov Cluster (MCL) [11] algorithm seems to be one of the most successful clustering procedures used in partitioning a PPI network into densely connected modules. In 2001, Enright et al. [11] used MCL to assign proteins into families based on precomputed sequence similarity information. Their results show that the method is ideally suited to the rapid and accurate detection of protein families on a large scale. Brohee and Helden [19] applied four algorithms, Molecular Complex Detection (MCODE) [12], Super Paramagnetic Clustering (SPC) [20], Restricted Neighborhood Search Clustering (RNSC) [21] and Markov Clustering (MCL) to six protein interaction networks obtained from high-throughput experiments and compared the resulting clusters with the annotated complexes. They found that the analysis of high-throughput data supported the superiority of MCL for the extraction of complexes from interaction networks. Vlasblom and Wodak [22] found that the advantage of MCL over a number of other procedures which were specifically designed for partitioning protein interactions graphs was dramatic for unweighted protein interaction graphs. Their experimental results show that the MCL procedure is significantly more tolerant to noise and behaves more robustly than the other algorithms. For MCL algorithm, the inflation parameter can be set as different values. Wu *et al*. [23] concluded that 1.9 was the best inflation parameter for the DIP data. Our experimental results show that the optimal inflation parameter is 2.0 when the MCL algorithm is applied to the yeast PPI network. MCL thus remains the method of choice for identifying protein functional modules from the TC-PINs. The following paragraph will briefly outline the principles of MCL. The MCL process consists of two operators called expansion and inflation. It involves changing the values of a transition matrix toward either 0 or 1 at each step in a random walk, until the stochastic condition is satisfied. The algorithm first adds self-loops to the input graph - by default, the loop weight for each node is assigned as the maximum weight of all edges connected to the node - and then translates this graph into a stochastic 'Markov' matrix. This matrix represents the transition probabilities between all pairs of nodes and the probability of a random walk of length *n *between any two nodes can be calculated by raising this matrix to the exponent *n*- a process referred to as expansion. The inflation step introduces a non-linearity into the process, in order to strengthen intra-cluster flow and weaken inter-cluster flow. Since greater path lengths are more common within clusters than between different clusters, the probabilities between nodes in the same module will typically be higher in expanded matrices. MCL further exaggerates this effect by taking entry wise exponents of the expanded matrix and then rescaling each column so that it remains stochastic. Iterating expansion and inflation will subdivide the PPI network into many segments as protein functional modules or complexes.

The MCL procedure is applied to create candidate functional modules from each TC-PIN. Then, a script program implemented using the Perl language is used to remove modules that include only one gene product or belong to another one. Redundant modules are also removed.

### Evaluation metrics

#### Pseudorandom network

A problem of evaluation is that a certain proportion of interacting proteins can be assigned to the same modules by chance. In order to estimate the random expectation of correct grouping, NetworkAnalyzer (http://www.mpi-inf.mpg.de/) is used to preserve the connectivity of each node, while edges are reallocated at random to build a pseudorandom network of the same size (consisting of the same number of nodes and edges) as static yeast PPI networks.

#### Matching evaluation

Comparing predicted modules with known ones is a common method of evaluation. For many years, the yeast protein modules catalogued by the Munich Information Center of Protein Sequences (MIPS) database have been widely used to generate protein-protein interaction reference sets. Although this catalogue has served the community very well, it no longer reflects the current state of knowledge in the field. We derive 408 typical modules including two or more proteins each from the CYC2008 [24] as the benchmark module set and use the same scoring scheme used by [12] to determine how effectively a predicted module matches a known module. If two complexes overlap each other, they must share one or more proteins. The Overlap Score (*OS *) of a predicted module vs. a benchmark module is then a measure of biological significance of the prediction, assuming that the benchmark set of modules is biologically relevant. The overlap score between a predicted and a known module is calculated by using(1)

where, *i *refers to the number of proteins shared by a predicted module and a benchmark module, *g *is the number of proteins in the predicted module and *h *is the number of proteins in the benchmark module. If OS (Overlap Score) is 1, it means that a predicted module has the same proteins as a benchmark module. On the contrary, when OS equals to 0, there is not a shared protein between the predicted module and the benchmark module [12].

Defining the number of true positives (*TP*) as the number of MCL predicted modules with *OS *over a threshold value and the number of false positives (*FP*) as the total number of predicted MCL modules minus *TP*. The number of false negatives (*FN*) equals the number of known benchmark modules not matched by predicted modules. Sensitivity is defined as [*TP/(TP+FN)*] and specificity is defined as [*TP/(TP+FP)*] [12]. *f-measure*, or the harmonic mean of sensitivity and specificity, can then be used to evaluate the overall performance [8]:(2)

#### Statistical evaluation

The Gene Ontology project provides an ontology of defined terms representing gene product properties. The ontology covers three domains: Cellular Component (*CC*), the parts of a cell or its extracellular environment; Molecular Function (*MF *), the elemental activities of a gene product at the molecular level, such as binding or catalysis; and Biological Process (*BP*), operations or sets of molecular events with a defined beginning and end, pertinent to the functioning of integrated living units: cells, tissues, organs and organisms. The GO ontology is structured as a directed acyclic graph and each term has defined relationships to one or more other terms in the same domain and sometimes to other domains. The GO vocabulary is designed to be species-neutral and includes terms applicable to prokaryotes and eukaryotes, single and multicellular organisms.

A module is associated with a known function by determining whether the number of proteins known to be annotated with the function is enriched, as judged by the hypergeometric distribution. The P-value can be used to determine the probability that a given set of proteins is enriched by a given functional group by random chance. In [25], it is used as a criterion to assign each cluster to a known function. The smaller the P-value, the more evidence the clustering is not random. In terms of GO annotations, a group of genes with a smaller P-value is more significant than the one with a higher P-value.

Consider a cluster of size *c*, with *m *proteins sharing a particular annotation *A*. Also assume that there are *N *proteins in the PPI database, and *M *of them are known to have annotation *A*. Given that, the probability of observing *m *or more proteins that are annotated with *A *out of *N *proteins is:(3)

Based on above formulation, a P-value is calculated for each of three ontologies. In the case of multiple annotations from the same ontology, the one with the smaller P-value is assigned to the cluster as functional annotation. That being said, the P-value without any restriction is not enough to label clusters as significant. Hence we use the recommended cutoff value of 0.01 [26] in order to select significant modules within each ontology.

A popular software package for evaluating the statistical significance of GO terms represented in a set of genes extracted from a population is GO::TermFinder, which calculates P-values using formula (3) [27]. GO::TermFinder accepts a list of genes of interest and returns a list of GO terms with which the genes are associated, with corresponding P-values and FDR values (if desired) associated with the enrichment of these terms in the gene list. In this research, the direct use of GO TermFinder is not convenient for analyzing GO enrichment of more than 2000 modules uncovered from the TC-PINs, because this software package can only handle one module at a time. Therefore, combined with the latest version of this toolkit [28], we have used the Perl language to develop a procedure that can automatically process a large number of functional modules in turn.

## Results and Discussions

### Functional modules in various networks

**Figure 2:**
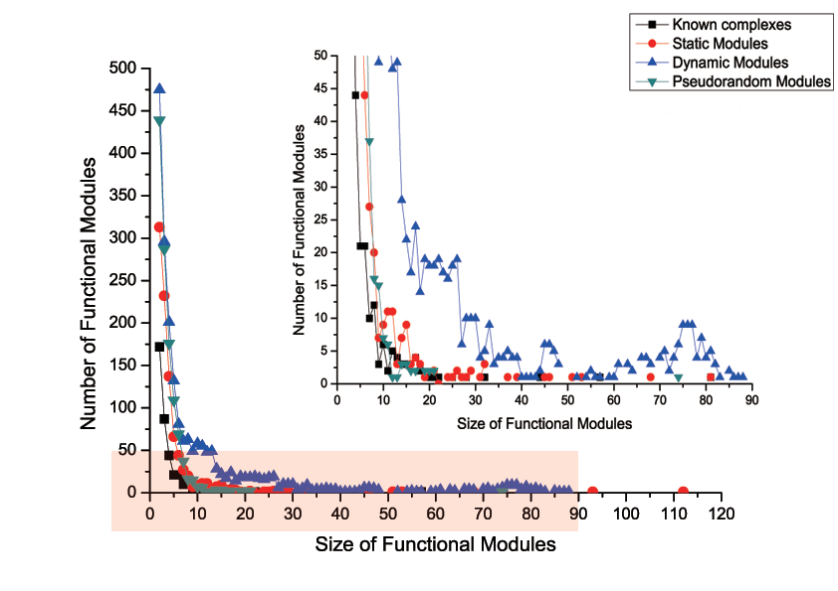
**The distribution of module sizes for various networks compared to the CYC2008 data**. Figure 2 is developed with the number of functional modules as a vertical coordinate, the size of the modules as a horizontal coordinate.

The MCL procedure is applied to three kinds of networks, namely, the pseudorandom network, the static PPI network and the TC-PINs, to detect functional modules. Table 1 shows the properties of functional modules identified from different sources. The second column in this table refers to the number of functional modules predicted from various networks. The modules consist of 2 or more proteins. The number of modules in the TC-PINs is over twice the number of modules identified from the static PPI network. More significantly, the largest module of the TC-PINs is smaller than that of the static PPI network. Generally, the predicted complexes with small size tend to have large P-values. Meanwhile, the predicted complexes with big size have low P-values [29]. Therefore, the predicted complexes with big size are investigated further. Figure 2 shows the distribution of module sizes for various networks compared to the CYC2008 data (the known complex set). The rectangular region is enlarged at the upper right of center. As shown in Figure 2, the number of the big modules detected from the TC-PINs is much greater than that of the big modules predicted from the static PPI network. For example, only 3 modules containing 80 or more proteins are derived from static PPI network, but the TC-PINs provide 18 modules of size≥80. The results (Additional file 1) show that 12 of 18 functional modules consisting of 80 or more proteins have annotation in the GO biological process category. So the functional modules identified from the TC-PINs are much more specific than the complexes from the static PPI network, and the TC-PINs can at least partly avoid false positives. Thus, it is more reasonable to detect functional modules from the TC-PINs than from a static PPI network. Certainly, the experimental results of pseudorandom network prove that the TC-PINs have biological meaning. Features of functional modules of the TC-PINs demonstrate partially that the reconstruction of dynamic networks is successful. But this is not enough. Therefore, more strict and thorough comparative analysis is performed later.

**Table 1:** The properties of functional modules predicted from various networks.

	#Modules	Max. size	Avg. size
TC-PINs	2,063	88	12.32
Static PPI network	932	112	5.04
Pseudorandom network	1,169	74	3.82

Table 1 gives The number, maximum size and average size of the modules generated by the MCL procedure in three different kinds of networks.

### Comparison with the known modules

The validation and analysis of the modules derived from different types of networks involves comparison with prior knowledge. In this subsection, the modules obtained from these networks are compared with the modules annotated in the CYC2008, by computing the same statistics as described above [24]. Table 2 shows the comparison results of identified modules from the TC-PINs, static PPI network and pseudorandom network, respectively.

**Table 2:** The results of various networks.

	TC-PINs		Static PPI network		Pseudorandom network	
	
	Mp	Mk	Mp	Mk	Mp	Mk
*OS *≥ 0.0	408	408	408	408	408	408
*OS *≥ 0.1	757	331	291	307	262	222
*OS *≥ 0.2	443	232	175	197	38	41
*OS *≥ 0.3	290	159	131	142	0	0
*OS *≥ 0*:*4	187	125	102	109	0	0
*OS *≥ 0.5	135	98	84	87	0	0
*OS *≥ 0.6	76	63	47	48	0	0
*OS *≥ 0.7	31	28	25	25	0	0
*OS *≥ 0.8	20	20	18	18	0	0
*OS *≥ 0.9	18	18	15	15	0	0
*OS *= 1.0	18	18	15	15	0	0

In Table 2, Mp is the number of correct predictions which match at least a real complex and Mk is the number of real complexes that match at least a predicted functional module.

The number of predicted modules is shown against the number of matched known modules over a range of *OS *thresholds from threshold of 0 to 1.0 (in 0.1 increments). Threshold of 0 means that a predicted module need not share any proteins with a known module to be considered a match. That is, all modules in three different networks can perfectly match all of the 408 benchmark modules with *OS *= 0.

Table 2 indicates that the matched results from the TC-PINs are considerably better than those from the static PPI network-not to mention the pseudorandom network. In [12], Bader *et al*. research the effect of Overlap Score threshold on number of predicted and matched known complexes and find that the average and maximum number of matched known complexes drops more quickly from zero until an *OS *threshold of 0.2 than from 0.2 to 0.9 indicating that many predicted complexes only have one or a few proteins that overlap with known complexes. An *OS *threshold value which falls within the region from 0.2 to 0.3 thus seems to filter out most predicted complexes that have insignificant overlap with known complexes. In Table 2, Mp is the number of correct predictions which match at least a real complex and Mk is the number of real complexes that match at least a predicted functional module. As shown in Table 2, when *OS *= 0.2, out of 2063 functional modules predicted from the TC-PINs, 443 match 232 real complexes; but out of 932 complexes identified from the static PPI network, 175 match 197 real complexes. With *OS *= 0.3, out of 2063 functional modules predicted from the TC-PINs, 290 match 159 real complexes; but out of 932 complexes identified from the static PPI network, 131 match 142 real complexes. Next, the three types of evaluation metrics described earlier are used to evaluate the quality of the predicted modules. For the reason stated above, the typical value of 0.2 is chosen as the threshold to take the specificity, sensitivity and f-measure analysis.

**Figure 3:**
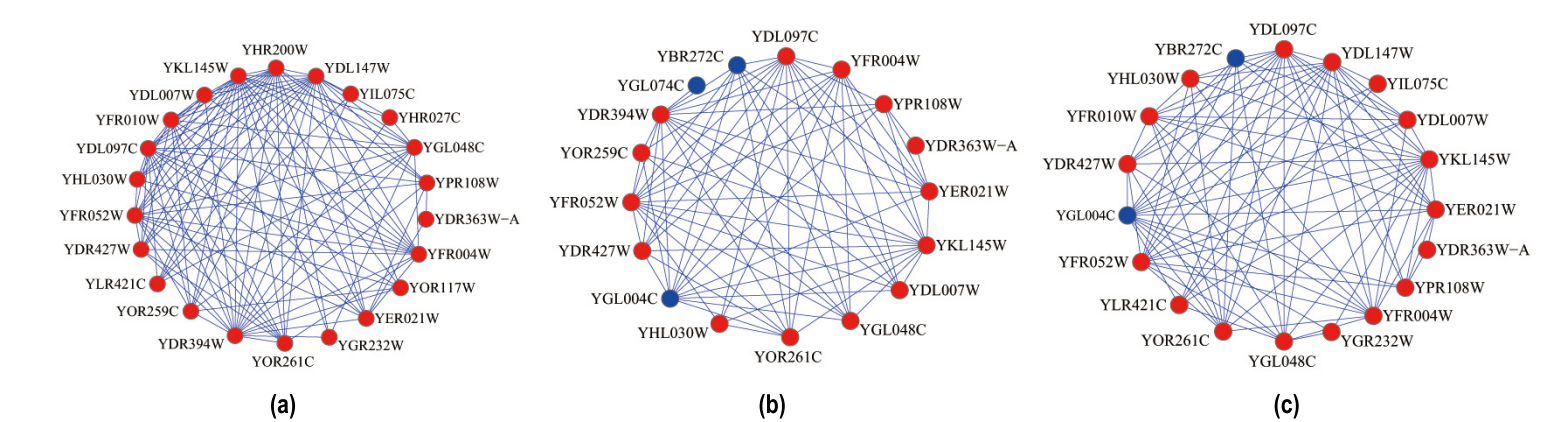
**The 19/22S regulator complex predicted from the static PPI network and the TC-PINs**. Figure 3(a) shows the real 19/22S regulator complex in the benchmark. Figure 3(b) and Figure 3(c) are the 19/22S regulator complex predicted from the static PPI network and the TC-PINs, respectively. For each predicted complex, the proteins in red color are involved in the real 19/22S regulator complex and those in blue color are not.

Figure 3 illustrates an example, in which the predicted 19/22S regulator functional module can cover more proteins in the real 19/22S regulator complex (GO: 0008541) [24]. In this example, the real 19/22S regulator complex (Figure 3(a)) in the benchmark consists of 22 proteins. The functional module predicted from the TC-PINs (Figure 3(c)) has 19 proteins and covers 17 proteins (in red color) of the real 19/22S regulator complex. Meanwhile, the complex identified from the static PPI network (Figure 3(b)) covers only 14 proteins of the real 19/22S regulator complex. Compared with a static PPI network, including 4,950 proteins and 21,788 interactions, each TC-PIN only consisting (on average) of about 3,520 proteins and 14,904 interactions or so(Additional file 2) is much smaller. Nevertheless, the number of modules matched perfectly in the TC-PINs is more than that of modules matched perfectly in static PPI network. Furthermore, the experimental results show that when the MCL procedure runs on TC-PINS, it has higher specificity, sensitivity and f-measure than the ones when it runs on the static PPI network, which is specifically given in Table 3.

**Table 3:** Comparison of the Sn(sensitivity), Sp(specificity) and f-measure of various networks.

	#Modules*	Avg.size	Perfect match	Sn	Sp	f-measure
TC-PINs	2,036	12.32	18	0.71	0.21	0.33
Static PPI Network	932	5.04	15	0.45	0.18	0.26
Pseudorandom Network	1,169	3.82	0	0.09	0.03	0.05

The performance comparison is presented in Table 3. We report the *sensitivity*, *specificity *and *F-measure*, with the threshold *OS *≥ 0.2.

Comparative analysis results in this subsection confirm that dynamic networks using temporal information (gene expressing profiles) improves our ability to discover biologically meaningful modules.

### GO enrichment analyses

In many studies, the GO has been used as the 'gold standard' to validate the functional relevance of the obtained network modules. In this subsection, as described by *GO enrichment analysis*, we used the GO biological process annotation, the GO molecular function annotation, and the GO cellular component to take GO enrichment analysis with the developed analytical tool based on GO::TermFinder software package [28].

First, we perform *BP*, *MF *and *CC *analysis of the functional modules discovered from the three different networks in an overall way. In our experiments, the P-values (with Bonferroni correction) of modules predicted from each kind of network are calculated by the tool, GO::TermFinder [28]. The functional modules predicted from the TC-PINs are considered to be significant, with corrected P-value≤0.01 [26]. The proportion of significant modules over all predicted ones and the average-log(P-value) of modules of size≥3 are listed in Table 4, 5 and 6. From Table 4, we can see that the proportion of significant modules extracted from the TC-PINs is up to 60.26%, while that of significant modules found in the static PPI network equals just 50.73%. The average -log(P-value) of modules identified from the TC-PINs is 7.20, but that of modules detected from the static PPI network reaches only up to 6.22. Out of 732 modules predicted from the pseudorandom network, only 62 are significant modules. The average -log(P-value) of modules discovered from the pseudorandom network is 2.17. The experimental results of these functional modules show that the pseudorandom network has little biological significance. The phenomenon reappears in Table 5. Above all, the GO CC analysis of modules discovered from the TC-PINs presents great strengths over that of modules identified from static PPI network in Table 6.

**Table 4:** BP analysis of modules identified from three kinds of networks.

	#Significant modules(*size *≥ 3)	#modules (*size *≥ 3)	Proportion of Significant modules	Average (-log(P-value))
TC-PINs	957	1588	60.26%	7.20
Static PPI Network	314	619	50.73%	6.22
Pseudorandom Network	62	732	8.33%	2.71

For biological process, the P-value of each module mined from each kind of network is calculated and the average -log(P-value) of modules of size ≥ 3 is listed in Table 4.

**Table 5:** MF analysis of modules identified from three kinds of networks.

	#Significant modules(*size *≥ 3)	#modules (*size *≥ 3)	Proportion of Significant modules	Average (-log(P-value))
TC-PINs	1,051	1588	66.18%	6.61
Static PPI Network	368	619	59.45%	6.12
Pseudorandom Network	239	732	32.65%	3.08

For molecular function, the P-value of each module mined from each kind of network is calculated and the average -log(P-value) of modules of size ≥ 3 is listed in Table 5.

**Table 6:** CC analysis of modules identified from three kinds of networks.

	#Significant modules(*size *≥ 3)	#modules (*size *≥ 3)	Proportion of Significant modules	Average (-log(P-value))
TC-PINs	868	1588	54.66%	11.00
Static PPI Network	256	619	41.36%	8.03
Pseudorandom Network	38	732	5.19%	3.03

For cell component, the P-value of each module mined from each kind of network is calculated and the average-log(P-value) of modules of size ≥ 3 is listed in Table 6.

**Figure 4:**
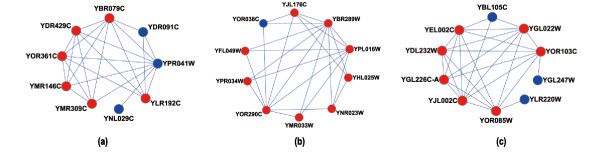
**Examples of functional modules predicted from the TC-PINs**. The predicted functional modules in Figure 4(a)-Figure 4(c) match eIF3 complex [24], SWI/SNF complex [24] and oligosaccharyl transferase complex [24], respectively.

Second, in order to carry out the finer comparison analysis of the three networks, the P-value range is divided into different intervals: *<*E-15, [E-15, E-10), [E-10, E-5), [E-5, 0.01) and ≥0.01. Table 7, corresponding to *BP*, Table 8, corresponding to *MF *and Table 9, corresponding to *CC*, show the number of detected modules whose P-values fall within one of the ranges: *<*E-15, [E-15, E-10), [E-10, E-5), [E-5, 0.01) and ≥0.01. In Table 7, 8 and 9, the number in parentheses indicates the proportion of the modules whose P-values fall within a given interval over all significant modules. As stated by *GO enrichment analysis*, the module whose P-value is more than 0.01 has no biological significance. Table 7, 8 and 9 show that there are more significant functional modules uncovered from the TC-PINs than from the static PPI network with P-value *<*1E-15, [E-15, E-10), or [E-10, E-5), in terms of both absolute number and percentage. For example, 1588 significant modules predicted from the TC-PINs, out of which 87 modules (5.48%) have a low P-value less than 1E-15 and 619 significant modules predicted from the static PPI network, out of which 18 modules (2.91%) fall within P-value*<*1E-15. Most important of all, this advantage is more obvious, particularly when the P-value is less than 1E-15, which would indicate the greatest biological significance. For the GO enrichment analysis: *BP *and *MF*, the absolute quantity of modules identified from the TC-PINs is more three times than that of the static PPI network and the percentage of modules found in the TC-PINs is almost twice as many as found by the static PPI network in this interval. For the *CC *analysis, the number of modules detected from the TC-PINs is much more five times that of the static PPI network and the ratio of the modules discovered from the TC-PINs exceeds that of the static PPI network by a factor of 2.5, with a P-value*<*1E-15. As shown in Table 7, 8 and 9, the P-values of most modules predicted from the pseudorandom network is greater than 0.01. For the pseudorandom network, this implies that there is virtually no biological meaning at all. In addition, Table 10 shows 10 functional modules with very low P-values, predicted from the TC-PINs. The False Discovery Rate (FDR) of every one of the 10 functional modules is 0.00%. The fifth column in Table 10 refers to the OS (Overlap Score) between the functional modules identified from the TC-PINs (in the third column) and real complexes(in the fourth column). The last column shows the number of proteins in the real complexes correctly covered by the functional modules predicted from the TC-PINs. In Table 10, proteins that have common GO annotation in the predicted functional module are in bold. Figure 4 gives three examples of functional modules predicted from the TC-PINs. The first example in Figure 4(a) is the functional module (ID = 6). It covers 6 out of 7 proteins in eIF3 complex(GO: 0005852) [24] and has three new proteins (in blue color). The predicted functional module in Figure 4(b) covers 9 proteins in SWI/SNF complex (GO: 0016514) [24] and has one novel protein (YOR038C) (ID = 9). The third example in Figure 4(c) is the predicted oligosaccharyl transferase complex (ID = 10), which covers 7 proteins in inoligosaccharyl transferase (GO: 0008250) [24] and has three new proteins (in blue color). Actually, there are many of the predicted functional modules matched well by the known complexes. Experimental results show that the biological significance of modules identified from the TC-PINs outperforms that of modules detected from the static PPI network.

**Table 7:** BP functional enrichment of the identified modules of size ≥3.

	*<*E-15	E-15 to E-10	E-10 to E-5	E-5 to 0.01	≥0.01
TC-PINs	87(5.48%)	142(8.94%)	257(16.18%)	471(29.66%)	631(39.74%)
Static PPI Network	18(2.91%)	29(4.68%)	77(12.44%)	190(30.69%)	305(49.28%)
Pseudorandom Network	0	0	1(0.14%)	61(8.33%)	670(91.53%)

Table 7 gives the results of BP functional enrichment and shows the percentage of detected modules whose P-values fall within one of the ranges: *<*E-15, [E-15, E-10), [E-10, E-5), [E-5, 0.01) and ≥0.01.

**Table 8:** MF functional enrichment of the identified modules of size ≥3.

	*<*E-15	E-15 to E-10	E-10 to E-5	E-5 to 0.01	≥0.01
TC-PINs	71(4.47%)	94(5.92%)	228(14.36%)	658(41.44%)	537(33.81%)
Static PPI Network	16(2.58%)	20(3.23%)	67(10.82%)	265(42.81%)	251(40.56%)
Pseudorandom Network	0	0	1(0.14%)	238(32.51%)	493(67.35%)

Table 8 gives the results of MF functional enrichment and shows the percentage of detected modules whose P-values fall within one of the ranges: *<*E-15, [E-15, E-10), [E-10, E-5), [E-5, 0.01) and ≥0.01.

**Table 9:** CC functional enrichment of the identified modules of size ≥3.

	*<*E-15	E-15 to E-10	E-10 to E-5	E-5 to 0.01	≥0.01
TC-PINs	203(12.78%)	104(6.55%)	201(12.66%)	360(22.67%)	720(45.34%)
Static PPI Network	31(5.01%)	22(3.55%)	73(11.79%)	130(21.00%)	363(58.65%)
Pseudorandom Network	0	1(0.14%)	1(0.14%)	36(4.92%)	694(94.81%)

Table 9 gives the results of CC functional enrichment and shows the percentage of detected modules whose P-values fall within one of the ranges: *<*E-15, [E-15, E-10), [E-10, E-5), [E-5, 0.01) and ≥0.01.

**Table 10:** Selected functional modules predicted from the TC-PINs and their P-values.

ID	Corrected P-values	Predicted functional modules	Real proteins complexes	OS	#common proteins
1	2.71e-36	**YNL151C YJL011C YOR116C YNL113W YNR003C YOR224C YPR187W YPR110C YOR207C YDL150W YDR045C YPR190C YKR025W YBR154C YKL144C **YOR341W YBR150C YDL164C YDR200C YBL015W YKL103C YKL218C YFR040W YPR067W YOR210W YLL019C YNL248C YPL150W	DNA-directed RNA polymerase III complex	0.54	16
2	5.58e-29	**YOR179C YJR093C YLR115W YKL018W YNL222W YAL043C YDR228C YNL317W YKL059C YDR195W YER133W YDR301W YPR107C YGR156W YLR277C **YDR412W YMR260C YHR100C YJL033W YOR250C YKR002W YML030W YGL256W YOR227W	mRNA cleavage and polyadenylation specificity factor complex	0.63	15
3	4.64e-24	**YGL004C YFR004W YLR421C YDR363W-A YDL147W YKL145W YPR108W YFR052W YGR232W YOR261C YIL075C YGL048C YER021W YDR427W YDL097C YDL007W YFR010W YHL030W YBR272C YBL084C**	19/22S regulator complex	0.69	17
4	4.35e-22	**YFR036W YDL008W YKL022C YLR102C YDR118W YNL172W YOR249C**	anaphase- promoting complex	0.53	8
5	5.50e-17	**YLR166C YER008C YGL233W YIL068C YDR166C YBR102C YIL068C YPR055W **YMR002W	Exocyst complex	0.77	7
6	2.85e-15	**YLR192C YOR361C YMR309C YDR429C YBR079C YDR091C YMR146C YPR041W **YNL029C	eIF3 complex	0.57	6
7	9.60e-14	**YOR115C YDR246W YGR166W YBR254C YDR407C YKR068C YDR108W YML077W **YGR143W	TRAPP complex	0.71	8
8	1.15e-14	**YNR035C YKL013C YIL062C YLR370C YBR234C YJR065C **YPR019W YNL040W YDL029W YNL012W	Arp2/3 protein complex	0.70	7
9	2.79e-13	**YHL025W YMR033W YPR034W YJL176C YBR289W YOR290C YPL016W **YNR023W YFL049W YOR038C	SWI/SNF complex	0.68	9
10	3.45e-12	**YGL226C-A YDL232W YJL002C YOR103C YGL022W YOR085W YEL002C **YBL105C YGL247W YLR220W	oligosaccharyl transferase complex	0.54	7

Table 10 gives 10 functional modules with very low P-values, predicted from the TC-PINs.

### Periodic genes in predicted functional modules

**Figure 5:**
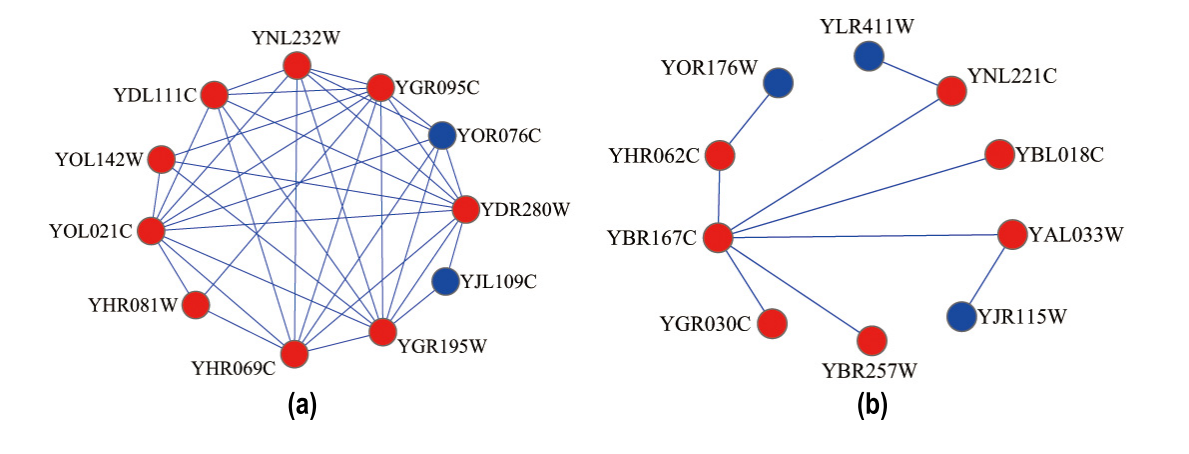
**Examples of functional modules including periodic genes**. The predicted functional modules in Figure 5(a) and Figure 5(b) match nuclear exosome complex [24] and nucleolar ribonuclease P complex [24], respectively. For each predicted functional module, the proteins in red color are involved in the real complexes and those in blue color are not.

Interestingly, there are 'special' proteins in many modules. The proteins don't cover any proteins in the real complex or don't share common function with other proteins. Ostensibly, they do not appear to be biologically significant. However, they are periodic gene products and perform a specific cellular function. Figure 5 presents two examples of functional modules including periodic genes. The predicted functional module in Figure 5(a) matches the nuclear exosome complex (GO: 0000176) [24]. It covers 9 out of 12 in the real complex. In addition, proteins YOL021C, YGR095C, YDR280W, YHR081W, YOL142W, YJL109C, YGR195W, YHR069C, YDL111C, YNL232W exactly share the same GO annotations in this module. It is very surprising that the protein YOR076C (in blue color) in this module doesn't share the same GO annotation and isn't involved in the real complex. But YOR076C is one of the most periodic genes and its expression peaks in the R/C (reductive/charging) metabolism [3]. At the same time, the protein YJL109C (in blue color) in this predicted module isn't involved in the real complex, but it also is periodic gene product and its expression peaks in the Ox (oxidative) metabolism [3]. The other example in Figure 5(b) is our predicted nucleolar ribonuclease P functional module (GO: 0005655) [24], which covers 7 out of 9 proteins in the real complex. The proteins YLR411W and YOR176W (in blue color) in this module don't share the same GO annotation and aren't involved in the real complex. However, they are periodic gene products. YLR411W's expression peaks during the R/B (reductive/building) metabolism. YOR176W's expression peaks in the R/C (reductive/charging) metabolism. The evidences constitute proofs that our computational discovery is consistent with the current biological knowledge, indicating that some novel knowledge could be discovered by our proposed method. Of course, biological experiments are necessary for further validating.

### Effect of the threshold selection

There are two main factors related to the threshold selection: the number of selected periodic genes and the number of biologically significant functional modules predicted from the TC-PINs. Table 11 shows the effect of threshold selection on the number of selected periodic genes and predicted significant functional modules for OS (Overlap Score) = 0.2. As shown in Table 11, when the threshold value is 0.02, all 3552 periodic genes are selected. Meanwhile, the Mk, Mp and f-measure of the modules identified from the TC-PINs are similar to that of the modules detected from the static PPI network. In other words, there is not obvious difference concerning biological significance between the TC-PINs and the static PPI network. As the threshold rises, the number of the new functional modules (referred to as Mp) and the number of the matched real complexes (referred to as Mk) also begin to increase. But the number of the selected periodic genes gradually decreases. When the threshold value increases to 1.6, the number of the real complexes matched by the functional modules predicted from the TC-PINs is much less than that of the real complexes matched by the functional modules identified from the static PPI network. In this situation, out of 3552 periodic genes, only 2786 are selected. From Table 11, it can be found that when a threshold value falls within the region from 0.3 to 1.4, the modules predicted from the TC-PINs are much more biologically significant than the modules identified from the static PPI network. Besides, when a threshold value falls within the interval: [0.7, 1.0], there are not much difference among the experimental results in Table 11. Therefore, 0.7 is chosen as the threshold to filter the gene expression profiles in our study.

**Table 11:** The results of the TC-PINs corresponding to various threshold values and the static PPI network.

	#Periodic Genes	#Modules	Mp Mk f-measure		
TC-PINs Threshold = 2.0	2589	1777	343	173	0.29
TC-PINs Threshold = 1.6	2786	1939	386	183	0.30
TC-PINs Threshold = 1.4	2893	1960	397	203	0.31
TC-PINs Threshold = 1.2	3001	2060	448	218	0.33
TC-PINs Threshold = 1.0	3108	2042	463	226	0.35
TC-PINs Threshold = 0.9	3167	2114	451	224	0.33
TC-PINs Threshold = 0.7	3273	2063	443	232	0.33
TC-PINs Threshold = 0.5	3377	1765	345	222	0.30
TC-PINs Threshold = 0.3	3487	1558	290	210	0.28
TC-PINs Threshold = 0.02	3552	980	177	194	0.26
Static PPI network		932	175	197	0.26

The second column in Table 11 refers to the number of selected periodic genes over various threshold values. The third column shows the number of predicted modules which match at least a real complex. The fourth column indicates the number of the real complexes that match at least a predicted module. The last column is the f-measure values of the predicted modules.

## Conclusions

In this paper, the TC-PINs are reconstructed by incorporating gene expression profiles into a static PPI network in order to discover the new biologically significant functional modules. And then we employ the MCL procedure to predict functional modules from the TC-PINs. Moreover, a series of comparative analyses on the matching and GO functional enrichment are carried out. The results show that compared with the static PPI network, there are much more biologically significant functional modules identified from the TC-PINs.

**Figure 6:**
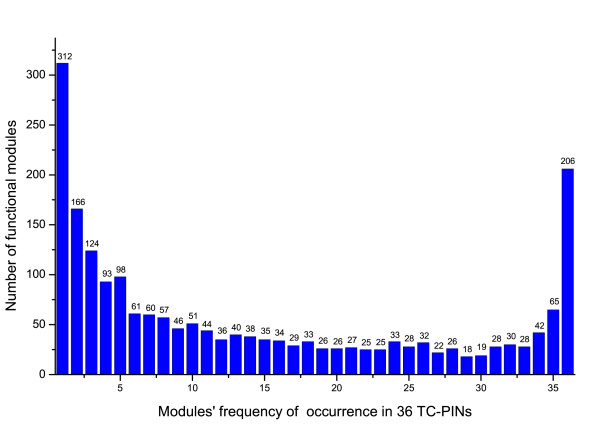
**The temporal specificity of the functional modules in the TC-PINs**. Figure 6 shows the temporal specificity of the functional modules identified from the TC-PINs created according to the threshold value of 0.7. It is developed with the number of functional modules as a vertical coordinate, the frequencies of occurrence of functional modules in 36 functional module sets as a horizontal coordinate.

When candidate functional modules are handled, we find that a functional module can be identified repeatedly from PPI networks at different time points. It seems to mean that functional modules are dynamic assemblies, which form in order to carry out specific functions in a temporal fashion. All functional modules identified from a TC-PIN at a certain time point form a set of functional modules. Since there are 36 TC-PINs, there are 36 sets of functional modules. In order to validate the temporal variability of functional modules over time points, the occurrence frequencies of functional modules in these 36 functional module sets are given in Figure 6. As shown in Figure 6, the intersection set of 36 functional module sets consists of 206 functional modules; 65 functional modules repeatedly appear 35 times in 36 functional module sets; likewise, 166 functional modules repeatedly only appear twice in 36 functional module sets. Moreover, 312 functional modules occur one time in 36 functional module sets. We think that it is a valuable work to research the temporal specificity of the functional modules discovered from the TC-PINs in future.

In addition, how to process the slightly different candidate functional modules is another problem worthy of research. A lot of functional modules are produced when the MCL algorithm runs on the TC-PINs. A few of these functional modules detected from the TC-PINs have probably the identical biological significance. Therefore, it is necessary to merge the functional modules. Certainly, it is easy to eliminate functional modules redundancy by discarding the modules whose all proteins belong to another module. Yet, merging the functional modules also creates a particular challenge: how to handle two modules sharing most but not all the proteins. Does the decision of whether or not to merge two slightly different modules depend on a small number of proteins not shared by the two modules? We believe that it will be valuable in the future to do an in-depth study of this problem.

In spite of the issues that still need to be resolved in the study of the TC-PINs, our research represents a successful, fundamental shift in the study of PPI from the static network to dynamic networks. Previous research on static PPI networks, such as identification of functional modules, protein function predictions, essential proteins and so on, can be performed on the TC-PINs and the resulting experimental results are much more satisfactory.

## Authors' contributions

XT carried out the TC-PINs reconstruction. XT, BL, ML and GC conceived of the study. BL and XT performed the data collection and analysis. XT drafted the manuscript under the guidance and supervision of JW and YP. All authors read and approved the final manuscript.

## Supplementary Material

Additional file 1 **GO Enrichment Analysis**. This RAR file is composed by 9 XLS files corresponding to GO enrichment (*BP*, *MF*, *CC*) analysis results from the TC-PINs, static PPI network and pseudorandom network, respectively. Click here for file

Additional file 2 **Table **1. This file presents topological analyses of the TC-PINs, the static PPI network and the pseudorandom network. NetworkAnalyzer(http://www.mpi-inf.mpg.de/) is used to compute eleven common topological parameters (number of nodes or proteins, number of edges or interactions, average number of neighbors, network density, network diameter, network heterogeneity, network centralization, characteristic path length, clustering coefficient, connected components, shortest paths) of the TC-PINs, static PPI network and pseudorandom network, respectively. The calculated results shown in Table 1 imply that although the size of each TC-PIN is smaller than static PPI network, these TC-PINs are still biologically meaningful protein interaction networks. Click here for file
